# Long-term trends and projections of ovarian cancer burden in China (1990 to 2040): an age-period-cohort analysis based on GBD 2021 data

**DOI:** 10.3389/fonc.2025.1652347

**Published:** 2025-08-14

**Authors:** Miaoling Huang, Meimei Guan, Qunxian Rao, Qing Chen, Jiating Wang, Zhongyi Fan, Jianpeng Xiao, Changhao Liu

**Affiliations:** ^1^ Department of Obstetrics and Gynecology, Sun Yat-sen Memorial Hospital, Sun Yat-sen University, Guangzhou, China; ^2^ Guangdong Provincial Institute of Public Health, Guangdong Provincial Center for Disease Control and Prevention, Guangzhou, China; ^3^ School of Public Health, Southern Medical University, Guangzhou, China

**Keywords:** ovarian cancer, age-period-cohort analysis, disease burden, trend projection, incidence, death, risk factors, disability-adjusted life years (DALY)

## Abstract

**Background:**

The growing burden of ovarian cancer is attracting widespread attention; the impact factors and the evolution trend of ovarian cancer burden need to be further studied.

**Methods:**

Ovarian cancer disease burden data for Chinese women were obtained from the Global Burden of Disease study 2021. We performed Age-Period-Cohort (APC) analysis to evaluate evolution trends across age, period, and cohort dimensions and identify contributing factors. Using the Bayesian Age-Period-Cohort (BAPC) model, we projected incidence and mortality trends through 2040.

**Results:**

In 2021, China recorded approximately 41,240 new ovarian cancer cases and 25,140 related deaths. From 1990 to 2021, age-standardized rates (ASRs) for incidence, mortality, and disability-adjusted life years fluctuated but increased steadily after 2015, with annual percentage changes of 1.6% (95%CI: 1.4%, 1.8%), 1.6% (95%CI: 1.4%, 1.9%), and 1.5% (95%CI: 1.3%, 1.6%), respectively. The APC model revealed a significant age effect with peak incidence occurring at 65–69 years; a period effect showing incidence and mortality rates resurged after 2015; and the cohort effects demonstrating bimodal incidence peaks in the birth cohorts of 1910–1914 and 1935–1939. Specifically, a 1% increase in the obesity rate was associated with a 3.06 (95%CI: 0.84, 5.28; *p* = 0.007) per 100,000 rise in ovarian cancer incidence. BAPC projections suggest that the ASRs of incidence and mortality of ovarian cancer in China will continue rising through 2040, possibly exceeding global trends.

**Conclusions:**

The burden of ovarian cancer in China remains significant; the increasing obesity rate in women may be a driver. The ovarian cancer burden has resurged in China since 2015, and it is projected to continue increasing by 2040.

## 1 Introduction

Ovarian cancer remains one of the most lethal gynecologic malignancies worldwide, characterized by its insidious onset, nonspecific symptoms, and frequent diagnosis at advanced stages, which collectively contribute to its poor prognosis. Globally, the burden of ovarian cancer accounts for approximately 314,000 new cases and 207,000 deaths in 2020 (1), and was projected to increase (2). The incidence rates of ovarian cancer have steadily risen in industrialized regions over the past decade, while geographic disparities highlight higher burdens in Europe and North America compared to Asia (2). However, China faces a substantial absolute disease burden of ovarian cancer, with its vast population base of over 1.4 billion, reporting nearly 61,100 new cases in 2022 (3). Recent advances in ovarian cancer diagnostics and therapy, including improved biomarkers, advanced imaging technologies, and targeted therapies like Poly (ADP-ribose) polymerase (PARP) inhibitors, have improved early detection and treatment outcomes (4). In China, high disease burden may be due to limited screening access, unequal availability of diagnostic tools (especially in rural areas), high costs of innovative treatments, and socioeconomic disparities (5). It was reported that a significant escalation in the burden of ovarian cancer in China has been observed from 1990 to 2019, as indicated by an upward trajectory in the incidence rate, mortality rate, and disability-adjusted life year (DALY) rate (5–7). The GBD 2021 dataset was officially released in May 2024 and uses the updated estimates and methodologies (8), hence it is necessary to update the predictive analysis on ovarian cancer burden in China to better address the healthcare and socioeconomic challenges.

The evaluation of ovarian cancer burden may be related to population aging, economic development, environmental change, and lifestyle change (6, 9, 10). A recent GBD-based analysis highlighting disparities in ovarian cancer profiles, risk factor exposure, and resource allocation across regions with varying economic development, underscores the need for region-specific analyses (11). Studies based on Asian women found an inverse association between parity and ovarian cancer risk (12). Conversely, large-scale case-control analysis found that obesity was significantly associated with the risk of ovarian cancer (13), which could increase metastasis and chemoresistance to ovarian cancer by altering macrophage polarization and fibrosis in the tumor microenvironment (14). However, research examining the influence of socioeconomic and demographic factors on the prevalence of ovarian cancer in China is currently limited.

Age-Period-Cohort (APC) analysis, is a well-established tool in epidemiological research for disentangling temporal drivers of disease patterns (15). The APC model allows us to separate the effects of age, calendar period, and birth cohort on disease incidence or mortality. Previous research has successfully applied the APC framework to analyze trends in various cancers, offering clear insights into the role of aging populations and changing environmental or lifestyle factors in shaping disease burdens (16, 17). Bayesian Age-Period-Cohort (BAPC) model extends traditional APC analysis by incorporating Bayesian statistical methods (18), which provide more flexible and robust estimates, especially in the presence of uncertainty and sparse data (19). The BAPC model has been increasingly used in cancer epidemiology due to its ability to capture complex temporal patterns and generate more reliable future projections compared to conventional approaches (20).

This study aims to explore the long-term trends in the burden of ovarian cancer among Chinese women over the past three decades and provide insights into future projection for up to 2040. We employed APC analyses to understand the effects of age, period, and cohort on ovarian cancer incidence and mortality, and investigated the contributions of birth rate and obesity rate to the ovarian cancer burden. Additionally, a BAPC model was conducted to project future trends up to 2040. These findings are intended to inform effective prevention and control strategies for ovarian cancer in China, ultimately contributing to a reduction in the disease burden.

## 2 Materials and methods

### 2.1 Data sources and inclusion criteria

#### 2.1.1 Ovarian cancer disease burden data

Data on ovarian cancer burden in China were extracted from the Global Burden of Disease Study 2021 (GBD 2021), curated by the Institute for Health Metrics and Evaluation (IHME) at the University of Washington (https://vizhub.healthdata.org/gbd-results). GBD 2021 generates disease burden estimates using a multi-step modeling pipeline, including the Cause of Death Ensemble Model (CODEm) for mortality, Spatiotemporal Gaussian Process Regression (STGPR) for incidence, and DisMod-MR 2.1 for prevalence and disability weights (8). The data used in this study were based on the following inclusion criteria.

Disease definition: Ovarian cancer was classified under the International Classification of Diseases (ICD), 11th Revision code (C56-C56.2 and C56.9) (11).

Metrics: Annual number of incident cases, deaths, and disability-adjusted life years (DALYs) for ovarian cancers, along with their corresponding age-standardized rates (ASRs), stratified by 5-year age groups (0–4 years to ≥85 years).

Temporal and Geographical Coverage: From 1990–2021, covering mainland China.

#### 2.1.2 Population data

Standardized population structure: Derived from the GBD 2021 world population age standard (21), used to calculate age-standardized rates (ASRs) for temporal and geographical comparability.

Population projections: Sourced from the United Nations’ “World Population Prospects 2022” (https://population.un.org/wpp), which provides annual population estimates (1950–2021) and projections (2022–2100) for 237 countries/regions, including China.

Population data stratified by 5-year age groups and sex, aligned with the age stratification of ovarian cancer burden data.

#### 2.1.3 Birth rate and obesity rate data

Birth rate data for China (1990–2021) were obtained from the National Bureau of Statistics of China (https://www.stats.gov.cn/) and incorporated into the APC model to evaluate demographic effects.

Age-standardized prevalence rates of obesity (BMI ≥30 kg/m²) among Chinese adult females (≥18 years) from 1990 to 2021 were extracted from the World Health Organization database (https://data.who.int/indicators) and incorporated into the APC model to examine the potential effects of obesity rate on ovarian cancer burden.

### 2.2 Statistical analysis

#### 2.2.1 Description analysis

The number of new cases and new deaths, age-standardized incidence rate (ASIR), age-standardized death rate (ASDR), and DALY rates of ovarian cancer in China from 1990 to 2021was analyzed. We estimated annual percentage change (EAPC) to quantify trends in ovarian cancer burden from 1990 to 2021. The EAPC was calculated based on a regression model fitted to the natural logarithm of the rate: ln(rate) = α + β*(calendar year) + ϵ, and then *EAPC = 100 ×[exp(β)—1]*, with its 95% confidence interval (CI) derived from the linear regression model (16). An age-standardized rate is deemed statistically increasing if both the EAPC estimate and its 95% CI are greater than 0. We further assessed the disease burden of ovarian cancer by age group in China in 1990, 2005 and 2021.

#### 2.2.2 Age-period-cohort analysis

APC analysis was used to disentangle observed trends to enable conclusions about the developments over three temporal dimensions: Age, representing the developments associated with chronological age over someone’s life cycle (17, 22). Period, representing the developments over calendar time which affect all age groups simultaneously. Cohort, representing the developments observed over different birth cohorts and generations.

We first draw a heatmap to facilitate the interpretation of temporal structures of the three APC dimensions for the ASIR and ASDR of ovarian cancer in China. Then, we estimated the effect of age, period, and cohort on disease burden by using the semi-parametric approach offered by generalized additive regression models (GAMs) according to Weigert et al.’s study (23).

To explore the possible socioeconomic and demographic factors influencing the evolution of ovarian cancer burden (24), the annual female obesity rate and birth rate in China with a lag of 0–10 years were included in the APC model. The effect was reported by coefficients of the regression model and its 95% CI.

#### 2.2.3 Epidemic trend projection

We predict the ASIR and ASDR of ovarian cancer from 2022 to 2040 by applying the BAPC model. BAPC uses integrated nested Laplace approximations (INLA) to approximate the posterior marginal distributions directly, without any Markov chain Monte Carlo sampling techniques, and therefore avoiding mixing and convergence issues (25). It helps researchers understand trends such as disease rate changes (20). Combining prior beliefs with observed data clarifies how age, period, and cohort impact outcomes while expressing the uncertainty in these estimates. The BAPC model used both sample information and prior information to obtain unique parameter estimates, which makes the results robust and reliable (26). The model can be represented as Equation (1):


(1) y(a,p,c) = α(a) + β(p) + γ(c) + ε(a,p,c)

where *y*(*a*, *p*, *c*)  is the incidence or mortality of ovarian cancer at a specific combination of age (*a*), period (*p*), and birth cohort (*c*); α(*a*), β(*p*), and γ(*c*) are the age, period (year), and cohort effects, respectively; and *ε*(*a*, *p*, *c*) is the residual error term.

We fitted the BAPC model based on the GBD data of ovarian cancer, GBD world population age standard and projected population from 2022 to 2040, and compared the projection results of China with those of the global. We assumed our predictions of ovarian cancer burden based on past trends, not considering changes in risk factors and interventions. To evaluate the performance of the projection model, we first used data from 1990 to 2011 to project data from 2012 to 2021. The mean absolute percentage error (MAPE) was calculated to assess the fitting of the projection model, and a value of less than 10% was used to determine a good fit (27).

In this study, we used “*APCtools*” and “mgcv” packages to conduct APC effect analysis. We built the BAPC models in the ‘*INLA*’ and ‘*BAPC*’ packages. All the analysis was performed in R software, version 4.2.0 (R Core Team, Vienna, Austria).

## 3 Results

### 3.1 Trend of ovarian cancer disease burden

**Figure 1:**
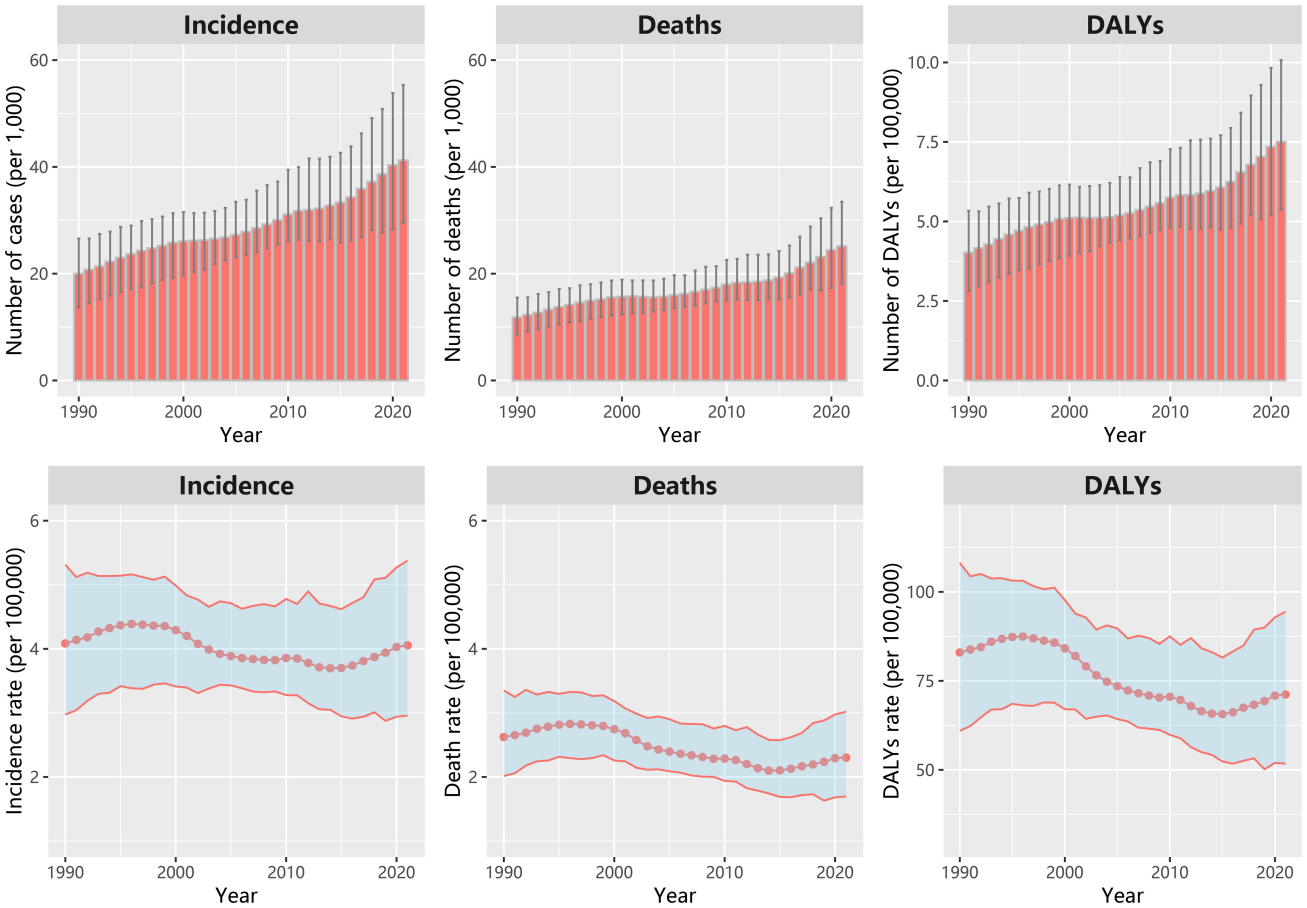
The number of new cases, deaths and DALYs; age-standardized incidence rate, deaths rate and DALYs rate of ovarian cancer in China from 1990 to 2021.

In China, there were about 41,240 new cases and 25,140 deaths owing to ovarian cancer in 2021 from GBD data. Between 1990 and 2021, the annual incidence, mortality, and DALYs attributable to ovarian cancer showed a consistent increase (**Figure 1**). The ASIR, ASDR and standardized DALYs rate for ovarian cancer have exhibited a wave change trend since 1990. These rates reached their zenith between 1995 and 1996, followed by a gradual decline. However, they have experienced a resurgence since 2015. The ASIR, ASDR in 2021 are 4.05 (95% CI: 2.96, 5.38) and 2.30 (95% CI: 1.70, 3.02) per 100,000 women, respectively (**Supplementary Table S1**).

The EAPC (1990-2021) for the ASIR, ASDR and standardized DALYs rate were -0.4% (95%CI: -0.6%, -0.3%), -1.0% (95%CI: -1.2%, -0.8%) and -1.0% (95%CI: -1.2%, -0.8%), respectively. While the EAPC for the ASIR, ASDR and standardized DALYs rate during 2015–2021 were 1.6% (95%CI: 1.4%, 1.8%), 1.6% (95%CI: 1.4%, 1.9%), 1.5% (95%CI: 1.3%, 1.6%), respectively. For the age group, the highest ASIR was found in the age group of 65–69 years in 2021, while the highest incidence rate was in the age group of 55–59 years in 1990. The ASDR increased with age, and it was a little lower in 2021 than that in 1990 and in 2005 for the aged 75+ years (**Supplementary Figure S1**).

### 3.2 Results of age-period-cohort effect analysis

**Figure 2:**
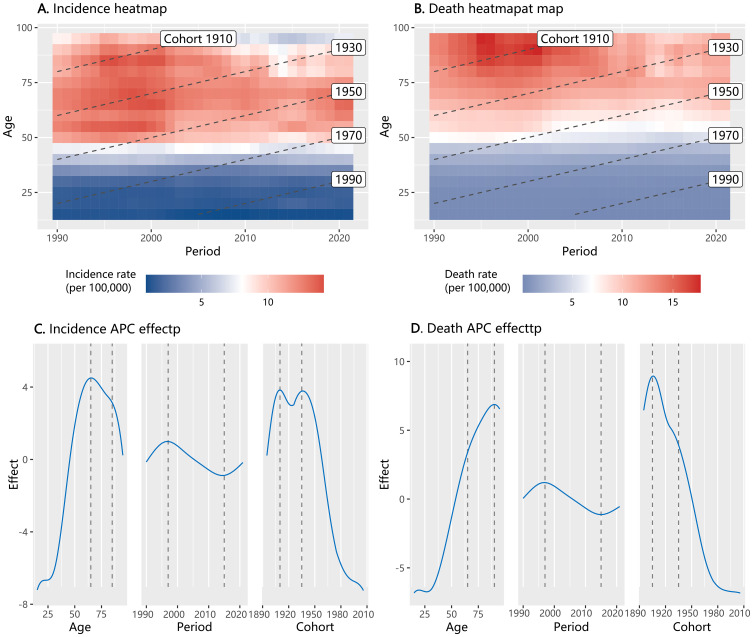
Heatmap of incidence and mortality of ovarian cancer by age, period, and cohort in China from 1990 to 2021, and the result of Age-Period-Cohort (APC) effect analysis (**A**: heatmap for incidence; **B**: heatmap for mortality; **C**: APC effect for incidence; **D**: APC effect for mortality).

The heatmap analysis of the age-period-cohort characteristics of ovarian cancer showed that both incidence and mortality rates varied across different age groups, calendar years, and birth cohorts (**Figures 2A–D**). In our APC effect analysis, we observed a significant rise in the age effect on ovarian cancer incidence rates starting from the age group of 30, with a peak observed in the 65–69 years age group. The impact of age on mortality rates also intensified with increasing age. Regarding the period effect, both incidence and mortality rates of ovarian cancer exhibited a peak from 1995 to 1997. These rates then experienced a decline but showed a resurgence from 2015. As for cohort effects, the incidence rate risk demonstrated two distinct peaks in the birth cohorts of 1910–1914 and 1935–1939. The cohort effect on ovarian cancer death rate reached a zenith in the birth cohorts of 1905–1909, after which it declined.

Demographic trends from 1990 to 2021 in China show persistently declining birth rates and steadily rising female obesity rates, with both trends exhibiting accelerated progression post-2010 (**Supplementary Figure S2**). In the analysis of the covariate effects within the APC analysis for ovarian cancer incidence, we found no statistically significant association between ovarian cancer incidence with the birth rate. However, the female obesity rate from the preceding decade appears to be a significant predictor of both the incidence and mortality of ovarian cancer. According to the APC single-factor model, the incidence of ovarian cancer may increase 3.06 (95% CI: 0.84, 5.28) per 100,000 for obesity rate increase by 1%. When the birth rate was included in the model, the effect value of the obesity rate ranged from 3.08 to 3.65 (*p* < 0.05). Similar findings were observed in the analysis of ovarian cancer mortality (**Table 1**).

**Table 1:** The effects of birth rate and female obesity rate estimated by the APC model.

Model	Incidence rate		Death rate	
	β (95%CI)	P value	β (95%CI)	P value
APC Model + single factor				
Birth rate	0.02 (-0.05,0.1)	0.521	0.04 (-0.02,0.11)	0.216
Birth rate_lag5	-0.01 (-0.07,0.05)	0.675	0 (-0.06,0.05)	0.928
Birth rate_lag10	0.01 (-0.04,0.07)	0.599	0 (-0.05,0.05)	0.953
Obesity rate	1.57 (-0.34,3.49)	0.109	1.50 (-0.70,3.70)	0.182
Obesity rate_lag5	0.94 (-2.34,4.22)	0.575	1.14 (-2.17,4.46)	0.501
Obesity rate_lag10	3.06 (0.84,5.28)	0.007	3.28 (1.11,5.45)	0.003
APC Model + two factors for the effect of Obesity rate_lag10				
+ Birth rate	3.65 (1.17,6.12)	0.004	4.07 (1.58,6.55)	0.002
+ Birth rate_lag5	3.08 (0.83,5.33)	0.008	3.32 (1.13,5.51)	0.003
+ Birth rate_lag10	3.23 (0.66,5.81)	0.014	3.35 (0.86,5.83)	0.009

### 3.3 Projection of ovarian cancer disease burden

**Figure 3:**
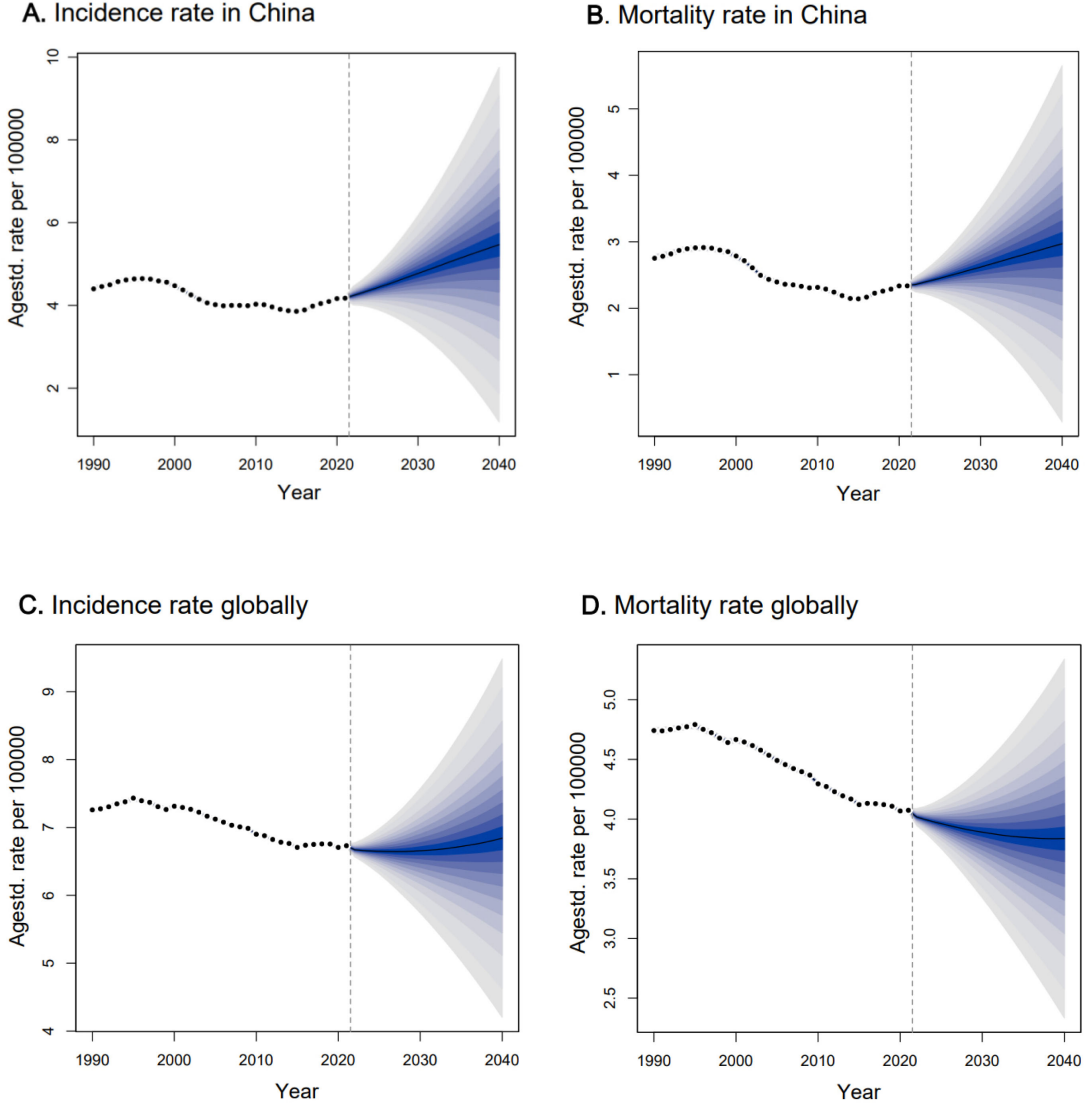
The projection trend for age-standardized incidence rate and age-tandardized mortality rate of ovarian cancer in China and globally from 2022 to 2040. Observed rates were shown as dots; the predictive mean was shown as solid line, together with the predictive distribution between the 5% and 95% quantiles, whereby the shaded bands show prediction intervals in increments of 5%, and the vertical dashed line indicates where prediction started.

The fitting result of the BAPC model projection from 2012 to 2021 showed that the MAPE for incidence and mortality fitting were 6.3% and 4.2% (**Supplementary Figure S3**), respectively, suggesting the model was ideal for the projection (27). Based on the model projection, we observed that the incidence and death rates of ovarian cancer would continue to increase in China if there was no additional intervention (**Table 2**; **Figures 3A, B**). The number of new cases in China would increase from 41,252 (95% CI: 40,701, 418,023) in 2021 to 53,911 (95% CI: 48,545, 59,277) in 2030 and 71925 (95% CI: 52,642, 91,207) in 2040, corresponding to the ASIR increasing from 4.08 (95% CI: 4.04, 4.12) per 100,000 women in 2021 to 4.65 (95% CI: 3.30, 6.00) per 100,000 in 2030 and 5.32 (95% CI: 1.20, 9.44) per 100,000 in 2040. Consequently, the number of deaths due to ovarian cancer would increase from 25,171 (95% CI: 24,752, 25,590) in 2021 to 34,894 (95% CI: 30,894, 38,894) in 2030 and 50,612 (95% CI: 33,976, 67,248) in 2040, corresponding to the ASDR increasing from 2.35 (95% CI: 2.33, 2.37) per 100,000 in 2021 to 2.63 (95% CI: 1.77, 3.49) per 100,000 in 2030 and 2.98 (95% CI: 0.29, 5.66) per 100,000 in 2040.

**Table 2:** The projection of incidence and mortality of ovarian cancer in China and globally.

Year	Incidence			Mortality		
	Number (95%CI)	Age-standardized rate (95%CI)	Change (%)*	Number (95%CI)	Age-standardized rate (95%CI)	Change (%)*
China						
2021	41252 (40701, 418023)	4.08 (4.04, 4.12)	–	25171 (24752, 25590)	2.35 (2.33, 2.37)	–
2030	53911 (48545, 59277)	4.65 (3.30, 6.00)	14.0	34894 (30894, 38894)	2.63 (1.77, 3.49)	11.9
2040	71925 (52642, 91207)	5.32 (1.20, 9.44)	30.4	50612 (33976, 67248)	2.98 (0.29, 5.66)	26.8
Globally						
2021	298869 (297426, 300312)	6.73 (6.71, 6.75)	–	185613 (184480, 186746)	4.07 (4.05, 4.09)	–
2030	350189 (335834, 364544)	6.66 (5.74, 7.58)	-1.0	219161 (209238, 229084)	3.89 (3.34, 4.44)	-4.4
2040	425348 (377006, 473689)	6.84 (4.17, 9.50)	1.6	268775 (235620, 301930)	3.84 (2.33, 5.35)	-5.6

*The age-standardized incidence rate or age-standardized death rate change by comparing with the rate in 2021.

When compared with the projection of the global ovarian cancer burden (**Figures 3C, D**), we observed the ASIR globally was relatively stable, with a slight increase after 2030, with an ASIR of 6.66 (95% CI: 5.74, 7.58) per 100,000 in 2030 and 6.84 (95% CI: 4.17, 9.50) per 100,000 in 2040. For the ASDR globally, it would decrease from 2022, with an ASDR of 3.89 (95% CI: 3.34, 4.44) per 100,000 in 2030 and 3.84 (95% CI: 2.33, 5.35) per 100,000 in 2040.

## 4 Discussion

The present study provides a comprehensive analysis of the trends in ovarian cancer burden among Chinese women, revealing a concerning trajectory of the disease. Although overall ASIR, ASDR and standardized DALYs rate have shown a slightly declining trend, the total numbers of new cases and deaths caused by ovarian cancer have increased, and the corresponding age-standardized rates have been steadily rising since 2015, particularly among obese women older than 30 years. The projected rises in ovarian cancer burden by 2040 are consistent with several published studies, highlighting the need for targeted interventions and preventive strategies to mitigate the ovarian cancer burden.

Our analysis and previous studies (5, 6) consistently found an increase in the numbers of incidence, death, and DALYs of ovarian cancer in China from 1990. The difference was that the age-standardized rate was found to increase continuously during 2000–2019 in previous studies, while our study showed that the ASIR, ASDR and standardized DALYs rate gradually recovered after 2015. The inconsistent results may be due to the algorithm optimization of the updated GBD 2021 database compared with the GBD 2019 version (8).

Per National Comprehensive Cancer Network (NCCN) Guidelines 2025 (28), ovarian cancer typically disseminates insidiously: low-grade lesions progress slowly, whereas high-grade tumors usually present at Stage III/IV. Standard therapy comprises R0 cytoreductive surgery followed by carboplatin–paclitaxel ± bevacizumab, with PARP maintenance for BRCA/HRD-positive disease. Five-year survival exceeds 90% in Stage IA but falls to ~30% in Stage IIIC/IV; subsequent management hinges on platinum-sensitive versus resistant relapse and clinical-trial access. These realities underscore that ovarian cancer remains formidable, emphasizing the imperative of risk-factor intervention, early detection, and precision treatment (29).

Several factors may have contributed to the increasing burden in China (1): Aging population: The elderly population in China is growing exponentially in the 21st century (30), and our analysis reflect that the elderly may be more susceptible to the attack of ovarian cancer (2). Lifestyle changes: Shifts in lifestyle, including dietary habits and reduced physical activity in China (31), have been linked to an increased risk of ovarian cancer (3). Reproductive Factors: Delayed childbearing and reduced parity in Chinamay increase the burden of ovarian cancer (32). (4) Lack of effective screening: The absence of a widely effective screening method for early detection of ovarian cancer leads to many cases being diagnosed at advanced stages (33) (5). Environmental exposures: Increasing industrialization and environmental pollution could lead to heightened exposure to endocrine-disrupting chemicals, which may contribute to ovarian cancer development (34) (6). Healthcare accessibility: Unequal access to healthcare, particularly in rural regions of China, can delay diagnosis and treatment, leading to poorer outcomes (35). Additionally, genetic mutations, particularly in the BRCA1 and BRCA2 genes, significantly increase the risk of ovarian cancer (36).

From the age group analysis, the highest incidence was found in the age group of 65–69 years in 2021, while the highest incidence was in the age group of 55–59 years in 1990, this study supported that the younger trend of ovarian cancer burden in China is not obvious (6), indicating a higher risk among postmenopausal women. Because hormone levels plummet in women after menopause, which is associated with an increased incidence of ovarian cancer (37, 38). With the aging of the population, reducing the ovarian cancer burden in postmenopausal women and older women should become the focus of ovarian cancer management strategies in China.

The APC analysis elucidates the complex interplay between age, periods, and birth cohorts. The age effect on ovarian cancer incidence and mortality rates increased with age and reached a peak in those aged 65–69 years, which was consistent with the result of age group analysis in this study. The period effect on ovarian cancer incidence rate showed a fluctuation curve, and an increasing trend since 2015, indicating that the period effect was an important factor in the evolution of ovarian cancer burden. The APC multiple factors analysis showed that the obesity rate was a significant risk factor for ovarian cancer incidence and mortality, with its prevalence among Chinese women increasing in tandem with the rise in ovarian cancer burden. Some studies have reported that obesity, especially central adiposity was a risk for ovarian cancer (24, 39, 40). A high body mass index indicates obesity, which could alter hormone levels and ovulation function (24). The increasing prevalence of overweight/obesity in China may have contributed to the rising burden of ovarian cancer (41). This finding aligns with global research emphasizing the role of obesity in cancer development, indicating a need for obesity management as part of ovarian cancer prevention strategies.

The BAPC model projections foresee a continued upward trend in both incidence and mortality rates of ovarian cancer by 2040 without intervention. Our findings were consistent with the previous projection that the burden of ovarian cancer will increase by 2030 in China (5, 6, 42). The upward trend in incidence and mortality rates may be related to the projected rapid population aging (43), changes in reproductive patterns, increased exposure to environmental risk factors, and increased prevalence of obesity and metabolic disorders (41, 44). Furthermore, when juxtaposed with global figures, we find that the projected trend of ovarian cancer burden in China has a higher rate than that globally, which means China may bear a substantial portion of the global ovarian cancer burden by 2040. Our results suggest the urgency of implementing public health policies to mitigate the disease burden. For instance, by optimizing screening methods, expanding the scale, and enhancing the ability for early diagnosis (45, 46), more patients with ovarian cancer can be identified and treated in the early stages of the disease. It is essential to strengthen health education on women’s reproductive systems, especially to increase the screening rate among the elderly, thereby effectively reducing the incidence and mortality of ovarian cancer.

In recent years, China has stepped up its fight against ovarian cancer, prioritizing early detection and prevention since 70% of cases are typically identified at later stages, where symptoms are more apparent but less treatable (6). The tumor suppressor genes BRCA1/2 play a critical role in maintaining genetic stability by repairing DNA, and mutations in these genes can significantly increase the likelihood of ovarian cancer (47). The Chinese healthcare system is now actively promoting genetic testing for BRCA1/2 mutations among women with a family history of ovarian cancer. Early identification through this method can lead to the prompt start of effective, targeted therapies. Additionally, some experts propose using a combination of serum CA125—a biomarker for epithelial cancer—and transvaginal ultrasound for early screening in women with a family history. In 2022, China implemented new quality control standards to streamline the diagnosis and treatment of primary ovarian cancer (48), aiming to enhance the overall quality of clinical treatment. With these advancements, there is a positive expectation for the future management and reduction of the ovarian cancer burden in China. To accelerate the reduction of the disease burden caused by ovarian cancer, some research work would prioritize (1) multicenter clinical trials of PARP inhibitors and immune-oncology combinations tailored to rBRCA-mutation profiles, (2) prospective validation of risk-adapted screening algorithms that incorporate HE4/CA-125 biomarkers and transvaginal ultrasound in high-risk populations, and (3) large-scale genomic–epidemiologic consortia to clarify gene–environment interactions (e.g., obesity, endocrine disruptors) unique to Chinese women.

Global experiences in ovarian cancer control provide referential insights for China’s response to the rising disease burden. The UKCTOCS trial demonstrated that multimodal screening which combines serum CA125 interpreted using the risk of ovarian cancer algorithm, transvaginal ultrasound and clinical assessment, improved early detection (49, 50), while South Korea’s risk-stratified approach (51, 52) and Japan’s comprehensive management practice of “surgery + chemotherapy + targeted maintenance therapy” (53) highlight the value of new treatment approach. Drawing upon these valuable international experiences and aligning with China’s progressive healthcare reforms, we suggest several strategic interventions that may help mitigate the anticipated disease burden. These approaches include biomarker-based screening with mHealth tools for high-risk groups, community-based healthcare policies under “Healthy China 2030” and targeted reproductive health education, specialist training for treatment improvement, and real-time cancer registry monitoring.

The strengths of our study lie in its use of robust statistical methods and comprehensive data from the GBD 2021, offering a nuanced understanding of the evolution of ovarian cancer in China. Some limitations should be mentioned. First, the potential biases inherent in the GBD data and the lack of granular regional data within China. Second, our study is subject to limitations inherent in the APC model, which assumes independence and additivity of age, period, and cohort effects, potentially overlooking complex interactions among these factors. Additionally, our analysis relies on historical data, which may not fully capture future trends due to changes in diagnostic practices, therapeutic advancements, and population dynamics. Third, data on the prevalence of obesity were retrieved from WHO estimates, which were defined as a BMI > 30 kg/m^2^, which may be inaccurate in Asian populations and perhaps lead us to underestimate the effects of obesity. Fourth, the potential impact of unforeseen events and evolving healthcare policies may alter the trajectory of ovarian cancer incidence and mortality in the future.

## 5 Conclusions

The incidence of ovarian cancer in China has been on a clear upward trajectory, with a notably steeper increase since 2015, and the increasing obesity rate in women may be a driver. Projections indicate that this trend will persist over the next two decades, with postmenopausal and older women being particularly at risk. This growing burden is a pressing concern that warrants immediate action. To effectively address this issue, it is imperative to bolster the management and preventive measures for ovarian cancer, such as disseminating screening methods, enhancing the quality of clinical diagnosis and treatment, advocating for a healthy lifestyle, and minimizing exposure to carcinogens, it is possible to reduce the incidence and mortality of ovarian cancer, thereby improving the health and well-being of women in China and around the world.

## Data Availability

Publicly available datasets were analyzed in this study. This data can be found here: https://vizhub.healthdata.org/gbd-results.
