# Supplementary material for: Long-term trends and projections of ovarian cancer burden in China (1990 to 2040): an age-period-cohort analysis based on GBD 2021 data
Source: Front Oncol. 2025 Aug 14;15:1652347. doi: 10.3389/fonc.2025.1652347 (PMC12390796; doi:10.3389/fonc.2025.1652347)
Supplement: Supplementary file 1 [file DataSheet1.pdf]

Long-term trends and projections of ovarian cancer burden in China (1990 to 2040): An age-period-cohort analysis based on GBD 2021 data

Supplementary materials

Table S1 The incidence and mortality of ovarian cancer in China from 1990 to 2021 (95% CI).

Figure S1 Incidence rate and death rate (per 100,000 women) of ovarian cancer by age groups in China in 1990, 2005 and 2021.

Figure S2 The change trend on the birth rate and women obesity rate in China from 1990 to 2021.

Figure S3 The fitting result of BAPC model projection from 2012 to 2021. (the orange circle is the age-standardized rate from 2012 to 2021, obtained from GBD 2021). Observed rates were shown as dots, together, the predictive mean was shown as solid line, together with the predictive distribution between the 5% and 95% quantile, whereby the shaded bands show prediction intervals in increments of 5%, and the vertical dashed line indicates where prediction started.

Table S1 The incidence and mortality of ovarian cancer in China from 1990 to 2021 (95% CI)

Year	Incidence				Mortality			
	Number of incidence (* 1000 women)	Cude rate (per 100,000 women)	Age-standardized Rate (per 100,000 women)	Proportion in woman cancer (%)	Number of deaths (*1000 women)	Cude rate (per 100,000 women)	Age-standardized Rate (per 100,000 women)	Proportion in woman cancer (%)
**1990**	20.00 (14.09, 6.19)	3.51 (2.47, 4.60)	4.08 (2.97, 5.31)	2.66	11.83 (8.95, 15.23)	2.08 (1.57, 2.67)	2.62 (2.02, 3.35)	2.03
**1995**	23.69 (18.02, 7.94)	3.98 (3.02, 4.69)	4.37 (3.42, 5.14)	2.83	14.19 (11.45, 6.59)	2.38 (1.92, 2.78)	2.82 (2.32, 3.30)	2.26
**2000**	26.08 (20.40, 0.38)	4.27 (3.34, 4.98)	4.29 (3.41, 4.99)	2.69	15.69 (12.81, 8.13)	2.57 (2.10, 2.97)	2.74 (2.26, 3.19)	2.30
**2005**	27.29 (24.05, 3.09)	4.33 (3.82, 5.25)	3.89 (3.43, 4.71)	2.38	15.98 (14.02, 9.39)	2.54 (2.23, 3.08)	2.40 (2.09, 2.90)	2.15
**2010**	31.10 (26.47, 8.62)	4.76 (4.05, 5.91)	3.86 (3.28, 4.78)	2.34	17.98 (15.23, 2.06)	2.75 (2.33, 3.38)	2.29 (1.94, 2.80)	2.35
**2015**	33.35 (26.55, 1.44)	4.95 (3.94, 6.15)	3.70 (2.95, 4.62)	2.21	19.29 (15.46, 3.57)	2.86 (2.29, 3.50)	2.10 (1.69, 2.57)	2.40
**2021**	41.24 (30.30, 4.55)	5.94 (4.36, 7.85)	4.05 (2.96, 5.38)	1.87	25.14 (18.53, 2.92)	3.62 (2.67, 4.74)	2.30 (1.70, 3.02)	2.50

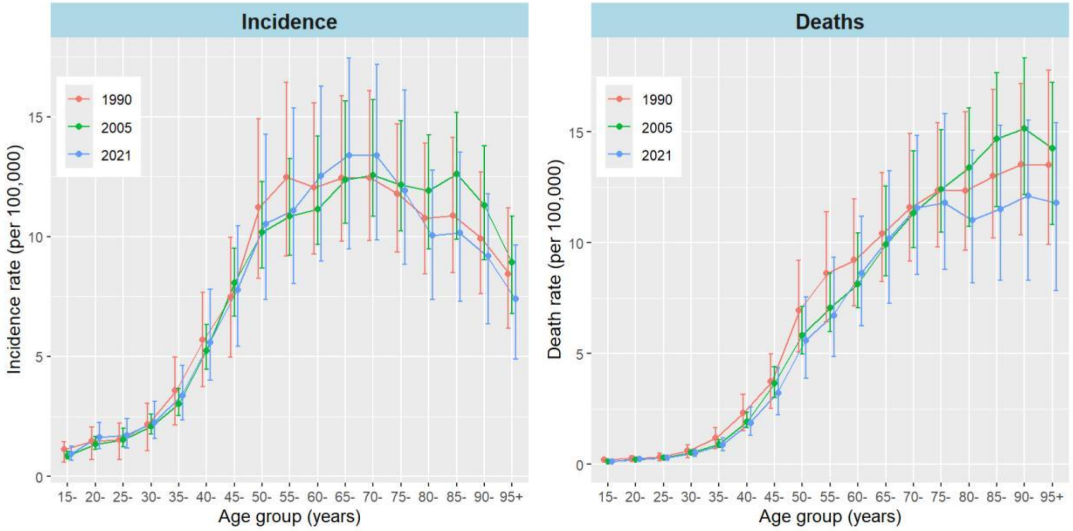

Figure S1 Incidence rate and death rate (per 100,000 women) of ovarian cancer by age groups in China in 1990, 2005 and 2021.

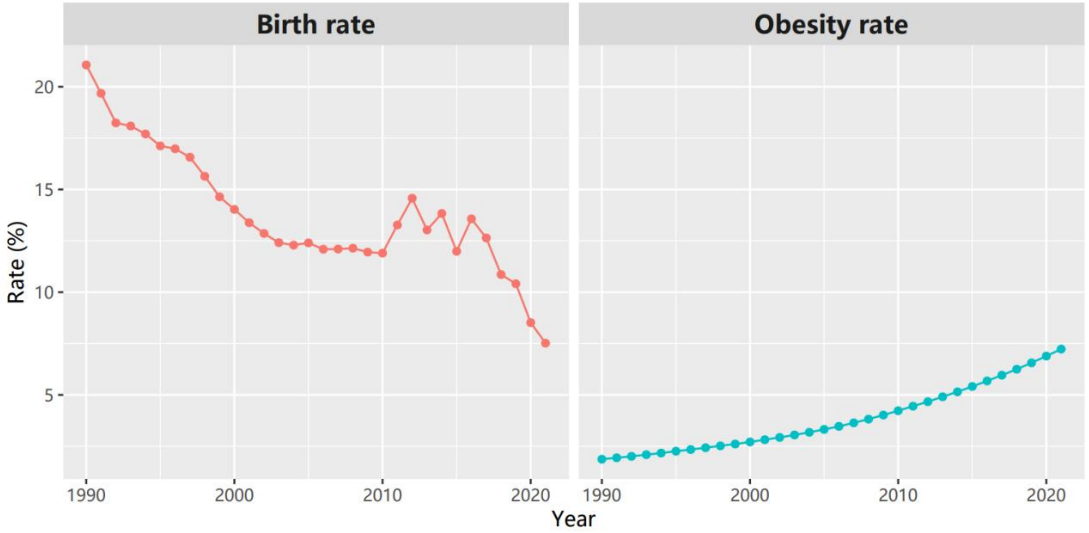

Figure S2 The change trend on the birth rate and women obesity rate in China from 1990 to 2021

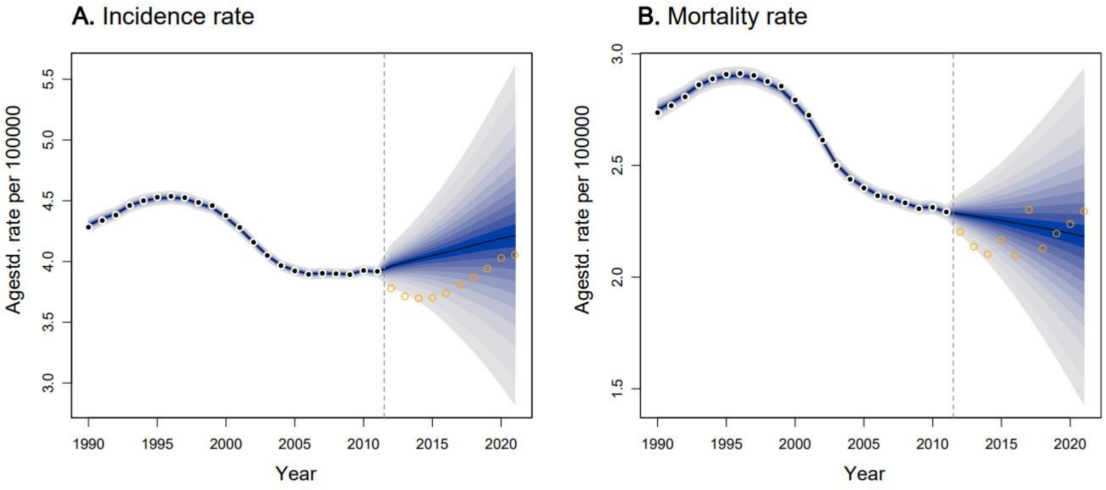

Figure S3 The fitting result of BAPC model projection from 2012 to 2021. (the orange circle is the age-standardized rate from 2012 to 2021, obtained from GBD 2021). Observed rates were shown as dots, together, the predictive mean was shown as solid line, together with the predictive distribution between the 5% and 95% quantile, whereby the shaded bands show prediction intervals in increments of 5%, and the vertical dashed line indicates where prediction started.
